# Hazardous air pollutants and breast cancer risk in California teachers: a cohort study

**DOI:** 10.1186/1476-069X-14-14

**Published:** 2015-01-30

**Authors:** Erika Garcia, Susan Hurley, David O Nelson, Andrew Hertz, Peggy Reynolds

**Affiliations:** Cancer Prevention Institute of California, Berkeley, CA 94704 USA; School of Public Health, University of California, Berkeley, CA 94720 USA; Division of Epidemiology, Department of Health Research and Policy, Stanford University School of Medicine, Stanford, CA 94305 USA

**Keywords:** Breast cancer, Hazardous air pollutant, Air pollution, National-scale Air Toxics Assessment, Estrogen receptor, Progesterone receptor, Ethylidene dichloride, Vinyl chloride, Benzene, Carbon tetrachloride

## Abstract

**Background:**

Studies suggest that higher breast cancer rates in urban areas persist after accounting for the prevalence of known risk factors, leading to speculation that urban environmental exposures, such as air pollution, may play a role in the etiology of breast cancer. Combining modeled ambient air concentrations with data from a large prospective cohort of California women with over 15 years of follow-up, we examined the relationship between breast cancer incidence and modeled concentrations of air pollutants shown to be mammary gland carcinogens (MGCs).

**Methods:**

The study population of 112,378 California Teachers Study participants included 5,676 women diagnosed with invasive breast cancer. Modeled annual average ambient air concentrations of 24 MGCs from the U.S. Environmental Protection Agency were linked to participants’ addresses. Cox proportional hazards models were used to estimate hazard rate ratios and 95% confidence intervals associated with residential MGC levels. MGCs were examined individually and as a combined summary variable for all participants, in selected subsets, and by tumor hormone responsiveness.

**Results:**

Initial models yielded some evidence for increased risk for several compounds, including acrylamide, carbon tetrachloride, chloroprene, 4,4'-methylene bis(2-chloroaniline), propylene oxide, and vinyl chloride, but after adjustment for multiple comparisons, only results for propylene oxide and vinyl chloride remained statistically significant. In subset analyses, estrogen-receptor positive or progesterone-receptor positive (ER+/PR+) tumors were associated with higher ambient levels of acrylamide, benzidine, carbon tetrachloride, ethylidene dichloride, and vinyl chloride, while ER-/PR- tumors were associated with higher ambient levels of benzene. Interesting results for different compounds were observed within certain subsets of the population.

**Conclusion:**

While our initial models yielded several elevated risk estimates, after adjusting for multiple comparisons and breast cancer risk factors, most hazard ratios were no longer statistically significant. Our subset analyses, however, suggest that elevated risk may be associated with some compounds for certain subgroups of interest. A summary variable for all 24 MGCs did not offer any advantage over the models for individual compounds. Results must be interpreted cautiously, as estimated exposure was limited to modeled annual average ambient air concentrations, and could not account for other sources or routes other than inhalation.

**Electronic supplementary material:**

The online version of this article (doi:10.1186/1476-069X-14-14) contains supplementary material, which is available to authorized users.

## Background

Breast cancer is the leading cancer diagnosed among women in United States [1]. An estimated 232,670 incident cases of invasive breast cancer were diagnosed in 2014. Known breast cancer risk factors explain less than half of all cases [2], underscoring the need to identify new risk factors. One of the strongest predictors of breast cancer incidence is geographic location, with the highest rates observed in urbanized areas [3–6]. Although some of the excess breast cancer risk in urban areas is likely due to differences in lifestyle [3, 7, 8], studies have suggested that geographic differences remain after accounting for the prevalence of known risk factors [9–11]. This has led to the speculation that environmental exposures in the urban environment, such as air pollution, may play a role in the etiology of breast cancer [12–14]. Outdoor air pollution was recently classified as carcinogenic to humans, primarily based on epidemiologic and toxicologic evidence for lung cancer [15]. That breast cancer is of interest, however, is reflected in a report by Rudel et al. that identified numerous chemicals associated with increases in mammary gland tumors in animal studies that are also air pollutants [16].

Few studies have examined ambient air pollution exposure and risk of breast cancer incidence in human populations. Two case–control studies examined breast cancer risk and proxies of exposure (e.g., residential proximity to chemical facilities), and found suggestive evidence for an association, but these studies used crude estimates of exposure, and did not evaluate specific compounds or classes of compounds [17, 18]. An ecological study exploring the association between releases of certain industrial chemicals, including six chemicals and six metals, found positive associations for select compounds and breast cancer rates, but could not account for personal breast cancer risk factors [19]. Although there is some evidence from occupational studies of workers exposed to a variety of compounds that are also common air contaminants, the relevance of these findings to the general population is not known given the differences in magnitude of exposures [20–28].

The United States Environmental Protection Agency (EPA) has produced modeled ambient concentrations for hazardous air pollutants at the census tract level for the entire United States for select years since 1996 [29]. Combining these data with data from a prospective cohort of over 112,000 women in California with over 15 years of cancer follow-up, we examined the relationship between breast cancer incidence and census tract levels of modeled concentrations of ambient air pollutants shown to be mammary gland carcinogens.

## Methods

### Study population

The California Teacher Study (CTS) is a large on-going prospective cohort established in 1995–1996 when 133,479 active and retired female teachers and administrators enrolled in the California State Teachers Retirement System returned a completed baseline questionnaire. A full description of the CTS cohort is available elsewhere [30]. For the purposes of this analysis, we excluded women (in a hierarchical manner) who were not residing in California at baseline (n = 8,867); had an unknown history of prior cancer (n = 139); had a prior history of invasive or in situ breast cancer (n = 6,212); asked to be removed from the study after joining (n = 1); or had an address that could not be geocoded (n = 5,882). The resulting study population was comprised of 112,378 women.

Use of human subjects data in this study was reviewed by the Human Subjects Research Committees of the Cancer Prevention Institute of California, the City of Hope, the University of Southern California, the University of California at Irvine, and the California Health and Human Services Agency and found to be in compliance with their ethical standards as well as with the U.S. Code of Federal Regulations, Title 45, Part 46 on the Protection of Humans Subjects.

### Outcome assessment

The CTS cohort is followed annually for cancer diagnosis, death, and change of address. Annual linkage between the California Cancer Registry (CCR) and cohort membership is used to identify incident cancer cases. Modeled after the National Cancer Institute’s Surveillance, Epidemiology, and End Results Program, the CCR maintains high standards for data quality and completeness and is estimated to be 99% complete [31]. Mortality files, as well as reports from relatives, are used to ascertain date and cause of death. Changes of address are obtained by annual mailings, responses from participants, and linkage to the U.S. Postal Service National Change of Address database. For the present analysis, we defined a case as any woman diagnosed with invasive breast cancer (ICD-03 site codes C500-C509, excluding those with histology codes of 9050–9055, 9140, and 9590–9992) after the date she completed her baseline questionnaire through Dec 31, 2011.

### Personal risk factors

The baseline questionnaire collected information on personal breast cancer risk factors. These include: age (calculated from date of birth and date of baseline questionnaire completion), race/ethnicity, family history of breast cancer in a first degree relative, age at menarche, age at first full-term pregnancy, total lifetime breastfeeding months, menopausal status and hormone therapy use at baseline, physical activity (defined as the average number of hours per week of strenuous activity over lifetime), body mass index (BMI), current alcohol consumption, smoking status, and total pack-years of smoking. Menopausal status was derived at the time of the baseline questionnaire from responses to questions about menstrual periods, duration and timing of both estrogen and progestin therapy, age of respondent, and ages at reported surgeries, if relevant.

Address at the time of the baseline questionnaire (1995–1996) was used to derive neighborhood socioeconomic status (SES). Using 2000 U.S. Census block group, data were obtained on a variety of SES variables including: percentage of adults over age 25 years having completed a college degree or higher; percentage of adults without a high school degree; median family income, percentage of adults employed in managerial/professional occupations; and percentage of population below the poverty line. A principal components analysis was conducted to create a composite variable of SES based on the five individual variables described above. The loading of the first principal component, categorized into quintiles, was then used in the Cox regression models. This metric was originally developed for covariate adjustment in a prior study of breast cancer in the CTS in which it was found to be predictive [32].

### Exposure assessment

Estimated ambient concentrations of hazardous air pollutants (HAPs) were assigned to CTS participants using data from the United States Environmental Protection Agency (EPA). The EPA produces the National-Scale Air Toxics Assessment (NATA) to identify and prioritize air toxics with respect to their potential population health risks [29]. The first NATA was conducted based on 1996 emissions data, and has been produced for every three years since for up to 180 HAPs, with the latest report based on 2005 emissions. Details on the assessment methods are available elsewhere [33] and are briefly described here. The EPA models annual ambient HAP concentrations using the Assessment System for Population Exposure Nationwide (ASPEN) for area, on-road, and non-road emission sources, and the Human Exposure Model (HEM) for major emission sources for the 2002 assessment. Both ASPEN and HEM use dispersion models that incorporate emissions data from the National Emissions Inventory (e.g., stack height, exit velocity, emissions rate, etc.) and meteorological data to estimate average annual ambient air concentration at the census tract level for the entire United States, including Puerto Rico and the Virgin Islands.

We determined the census tracts participants resided in by using a geographic information system (ArcGIS v.10, ESRI, Redlands, CA, USA) to geocode their addresses at the time of the baseline questionnaire to a 2000 U.S. Census tract. We then assigned to participants the corresponding modeled annual average ambient concentrations for the HAPs available in NATA for that census tract to create a proxy of inhalation exposure to outdoor air contaminants of interest.

Because the NATA concentration estimates were not specifically designed for use in health studies, we previously evaluated the agreement between the NATA modeled data and available monitored data for 12 compounds in the state of California [34]. These analyses found that the concentration estimates from the 2002 and 2005 assessments tended to have the best agreement between the modeled and monitored concentrations. We chose to use the 2002 ambient air concentration estimates for this study because that year was approximately the mid-point of our follow-up period (1995–2011). We decided against combining multiple years of estimates due to inconsistent methodical approaches and temporal variations in the level of agreement between years of the assessments which could introduce exposure misclassification [34]. The 2002 NATA estimates were generally reflective of the estimates for 1996, 1999, and 2005. The median Spearman correlation coefficient comparing 2002 estimates to these other years were 0.69, 0.76, and 0.88, respectively, among HAPs selected for this study.

To choose a set of HAPs to focus on for these analyses we relied upon the work of Rudel et al. [16], which identified 216 compounds with toxicological data indicating increased mammary gland tumors in laboratory animals. We identified 37 compounds as both mammary gland carcinogens (MGCs) listed by Rudel at al. and available in the 2002 NATA model concentration data as potentially eligible for our risk analyses here. Of these 37 compounds, 13 were excluded from our analysis due to insufficient variability (nine because they had the same value for all census tracts in the state of California; and four because they had less than 25% non-zero values). This left 24 MGC compounds that were evaluated in the risk analysis (Additional file 1: Table S1).

### Data analysis

Follow-up time was calculated as the number of months (rounded to the nearest full month) between joining the cohort (i.e., the date the baseline questionnaire was completed) and either the date of invasive breast cancer diagnosis, the date of in situ breast cancer diagnosis, the date of death, the date the woman moved out of California for a period longer than four consecutive months, or December 31, 2011, whichever came first. In situ breast cancer cases were not included with invasive cases and were instead censored at the time of diagnosis.

Because the analysis examined the relationship between risk of breast cancer and numerous compounds of interest, we used two different methods for parameterizing exposure in our models. First we examined each compound individually, categorized into quintiles of concentration, without including exposure from any other compound in the model. Second, we created a summary variable of exposure to all compounds of interest for each participant to assess exposure to multiple pollutants. This was done as follows: 1) all concentrations of zero μg/m^3^ for a compound (estimates of no exposure) were replaced by the minimum non-zero value observed for that compound, divided by two, 2) given the skewed distributions of these compounds, concentrations were transformed using log base 10, 3) for each compound, the log-transformed values were standardized across all participants by (i) subtracting the mean of the compound from each value and then (ii) dividing the values by the standard deviation, thus producing a standardized concentration for each compound, and 4) a summary variable for each participant was calculated by summing the participant’s standardized concentrations across all compounds of interest. (Across all participants, the resulting summary variable for exposure to all 24 MGCs had a mean of zero and a standard deviation of 16.7.) Finally, the summary variable was categorized into quintiles of standardized concentration across all participants and a quintile assigned to each participant. Associated risks were then assessed using this overall estimate of MGC exposure. This method for summarizing exposure to the 24 MGC was chosen over a simple summation because the range of concentrations among these compounds varied dramatically and because the potency factor for each unit concentration was not assumed to be similar across the compounds. Among compounds with cancer slope factors, these range from 500 (mg/kg-day)^−1^ for benzidine to 0.0035 (mg/kg-day)^−1^ for methylene chloride underscoring the incomparability of risk for a unit concentration of exposure [35].

Breast cancer is thought to be a hormonally mediated and multifactorial disease in which the effects of a risk factor may be strongly influenced by the endogenous hormonal milieu of the host [36]. Consequently, we further examined the relation between MGC concentration estimates and breast cancer risk in a number of a priori selected subsets of participants based on their baseline characteristics. These subsets included menopausal status (premenopausal/perimenopausal and postmenopausal); hormone therapy (HT) use (none/past use and current HT use); and BMI (<25 and BMI ≥25). Additionally, we assessed risk by tumor hormone responsiveness, either as estrogen-receptor positive or progesterone-receptor positive (ER+ or PR+), or as estrogen-receptor negative and progesterone-receptor negative (ER- and PR-). For the 18 compounds with known or suspected exposure from tobacco smoke (Additional file 1: Table S1), we further examined this risk association among non-smokers to reduce exposure misclassification. To address potential exposure misclassification due to reliance on baseline address for exposure assignment, we evaluated breast cancer risk among those participants who had no record of moving from their baseline address during the follow-up period (non-movers).

Cox proportional hazard models were used to estimate hazard rate ratios (HR) and 95% confidence intervals (95% CI) associated with concentration estimates for the MGCs using age at start and end of follow-up (in days) to define time on study. No apparent violation of the underlying assumption of proportional hazards was detected. All models were stratified by age at baseline and adjusted either for race alone or for race and personal risk factors of interest (all variables mentioned in personal risk factors section, save menopausal status). Tests for trend were conducted by parameterizing exposure into quintiles, setting the value to the median for each quintile, modeling exposure as a continuous variable, and testing for non-zero slope using a likelihood ratio test. Lastly, for each compound, the p-values for each non-degenerative quintile HR were adjusted for multiple testing across the ten subsets using False Discovery Rates (i.e., four adjustments for a compound with all quintiles present). All analyses were performed in SAS 9.3 (SAS Institute, Cary, NC, USA).

## Results

### Population characteristics

Table 1 shows the characteristics of the 112,378 CTS cohort members, including 5,676 breast cancer cases, which were included in our analysis. Both breast cancer cases and non-cases were predominantly non-Hispanic white, never smokers, postmenopausal, had a BMI <25, and consumed <20 grams of alcohol per day at baseline. Those with a breast cancer diagnosis tended to be older (mean age at baseline 57 years, compared with 53 years), more likely to have reported a family history of breast cancer, and more likely to be classified as a non-mover, compared with non-cases. Most cancers among women in this analysis were diagnosed 2002 or later and were tumor hormone responsive positive.

**Table 1:** **Distribution of select baseline characteristics among invasive breast cancer (BC) cases and non-cases included in the present analysis [** ***n*** **(%)]**

Characteristics	Non-cases	BC cases
Total	106,702 (100)	5,676 (100)
**Race/ethnicity**		
White	91,831 (86.1)	5,061 (89.2)
Black	2,894 (2.7)	134 (2.4)
Hispanic	4,805 (4.5)	153 (2.7)
Asian/Pacific Islander	3,907 (3.7)	193 (3.4)
Other/Mixed	3,265 (3.1)	135 (2.4)
**Age group (years)**		
20-29	5,212 (4.9)	42 (0.7)
30-39	14,906 (14.0)	276 (4.9)
40-49	28,880 (27.1)	1,268 (22.3)
50-59	25,562 (24.0)	1,800 (31.7)
60-69	16,655 (15.6)	1,363 (24.0)
70-79	10,807 (10.1)	749 (13.2)
≥80	4,680 (4.4)	178 (3.1)
**Family history of breast cancer (first degree relative)**		
Yes	12,221 (11.5)	965 (17.0)
No	90,390 (84.7)	4,504 (79.4)
Missing/Adopted	4,091 (3.8)	207 (3.7)
**Age (years) at menarche**		
≤11	23,558 (22.1)	1,350 (23.8)
12-13	59,732 (56.0)	3,137 (55.3)
≥14	21,746 (20.4)	1,109 (19.5)
Missing/Never	1,666 (1.6)	80 (1.4)
**Age (years) at first full-term pregnancy**		
≤24	27,115 (25.4)	1,522 (26.8)
25-29	31,105 (29.2)	1,753 (30.9)
≥30	18,110 (17.0)	987 (17.4)
Nulliparous*	28,229 (26.5)	1,310 (23.1)
Missing	2,143 (2.0)	104 (1.8)
**Duration of breastfeeding (months)**		
Pregnant, no live births	6,248 (5.9)	272 (4.8)
Never	16,977 (15.9)	1,092 (19.2)
<6	18,615 (17.5)	1,030 (18.2)
6-11	14,378 (13.5)	769 (13.6)
≥12	25,978 (24.4)	1,356 (23.9)
Nulliparous	21,849 (20.5)	1,036 (18.3)
Missing	2,657 (2.5)	121 (2.1)
**BMI (mg/m** ^**2**^ **)**		
<25	62,747 (58.8)	3,123 (55.0)
25-29	25,091 (23.5)	1,517 (26.7)
≥30	14,206 (13.3)	794 (14.0)
Outlier/Unknown	4,658 (4.4)	242 (4.3)
**Physical activity (hours/week)**		
≤0.50	31,771 (29.8)	2,028 (35.7)
0.51-2.00	33,993 (31.9)	1,801 (31.7)
2.01-3.50	18,709 (17.5)	876 (15.4)
3.51-5.00	10,155 (9.5)	482 (8.5)
>5.00	11,295 (10.6)	438 (7.7)
Missing/Unknown	779 (0.7)	51 (0.9)
**Menopausal status**		
Premenopausal	44,158 (41.4)	1,405 (24.8)
Perimenopausal	2,140 (2.0)	143 (2.5)
Postmenopausal	51,819 (48.6)	3,604 (63.5)
Missing	8,585 (8.1)	524 (9.2)
**Hormone therapy use**		
Past/never	18,796 (17.6)	1,052 (18.5)
Current	27,566 (25.8)	2,204 (38.8)
Other (pre/peri)	60,340 (56.6)	2,420 (42.6)
**Alcohol consumption (g/day)**		
None	34,278 (32.1)	1,663 (29.3)
<20	58,433 (54.8)	3,139 (55.3)
≥20	8,084 (7.6)	594 (10.5)
Missing	5,907 (5.5)	280 (4.9)
**Smoking status**		
Never	71,010 (66.6)	3,338 (58.8)
Former	29,656 (27.8)	1,938 (34.1)
Current	5,349 (5.0)	351 (6.2)
Missing	687 (0.6)	49 (0.9)
**Total pack-years of smoking**		
Never smokers	71,010 (66.6)	3,338 (58.8)
≤10	17,257 (16.2)	988 (17.4)
11-20	5,961 (5.6)	416 (7.3)
21-30	3,491 (3.3)	280 (4.9)
≥31	5,182 (4.9)	417 (7.4)
Missing/Unknown	3,801 (3.6)	237 (4.2)
**Moving status**		
Non-mover	55,496 (52.0)	3,496 (61.6)
**Year of diagnosis**		
1995-2001	—	2,384 (42.0)
2002-2011	—	3,292 (58.0)
**Tumor hormone responsive**		
Estrogen-receptor positive (ER+)	—	4,293 (75.6)
Progesterone-receptor positive (PR+)	—	3,477 (61.3)
ER+ or PR+	—	4,352 (76.7)
ER- and PR-	—	704 (12.4)

*Includes nulliparous women who were 1) pregnant without a live birth or 2) whose breast feeding history is unknown.

### MGC concentrations

**Figure 1:**
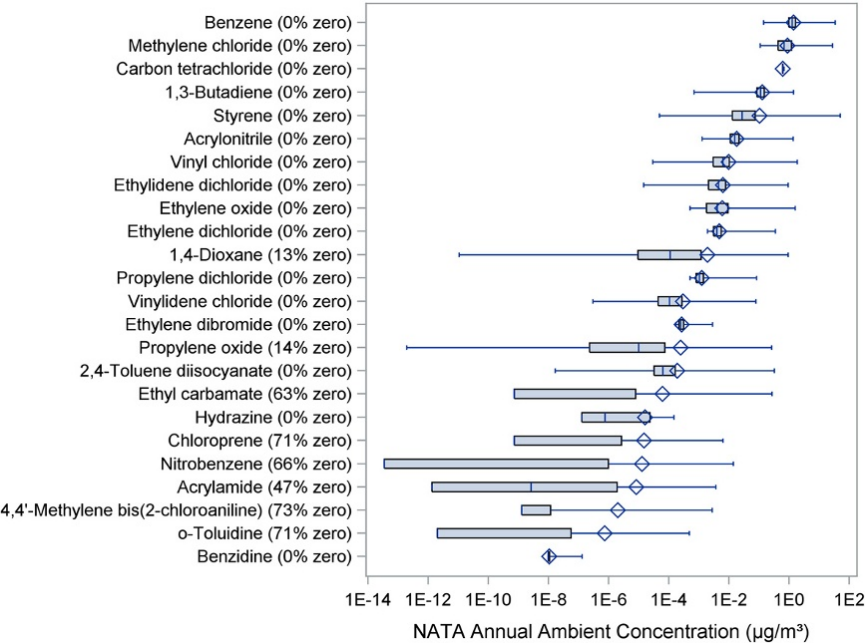
**Distribution of the 24 mammary gland carcinogen concentrations among the 112,378 female participants, including percentage of participants with concentration of zero.** Boxplot whiskers extend to minimum and maximum of data distribution. Zero values were set to the minimum non-zero value observed for the compound divided by two in order to be accommodated on the log scale.

The distribution of the 24 MGC concentrations among the CTS participants included in this study are presented in Figure 1. The distributions of these modeled annual average ambient concentrations varied widely. The means among the 24 compounds had a range of 8-orders of magnitude, from 1.40 μg/m^3^ for benzene to 1.07 × 10^−8^ μg/m^3^ for benzidine. Additionally, there was some variability in the range of concentrations within compounds, from the smallest range of 1.22 ×10^−7^ μg/m^3^ for benzidine to the largest range of 50.0 μg/m^3^ for styrene. While most compounds had no zero values, of the eight compounds that did, the percentage of zero values ranged from 13% to 73%.

### Risk analysis of individual MGC compounds

Results of the age-stratified and race-adjusted breast cancer incidence risk analysis for the individual compounds are shown in Table 2. For some compounds with a high percentage of zeros or another single value (acrylamide, benzidine, chloroprene, ethyl carbamate, hydrazine, 4,4'-methylene bis(2-chloroaniline), nitrobenzene, and o-toluidine), there are less than five exposure categories since all participants with the same value fell into a single quintile. The p-values from the tests for trend are displayed on the far right column. These initial models yielded some evidence of an increased risk for a number of compounds, including acrylamide, carbon tetrachloride, chloroprene, 4,4'-methylene bis(2-chloroaniline), propylene oxide, and vinyl chloride), with hazard rate ratios (HRs) for some quintiles and/or tests for trend statistically elevated at p < 0.05. After adjustment for multiple comparisons, however, only results for propylene oxide and vinyl chloride remained statistically significant (asterisk denotes significance after adjustment). Both compounds presented an inverted-U exposure-response relation with women in the third quintile of exposure having an approximate 11 to 12% increased risk of breast cancer. Further adjusting the models for breast cancer risk factors yielded similar patterns of risk for these two compounds but the HRs and tests for trend were no longer statistically significant (data not shown).

**Table 2:** **HRs (95**% **CIs) for breast cancer incidence (n = 5,676) by quintile of estimated exposure for individual mammary gland carcinogenic compounds and summary MGC variable**

	Quintile of concentration					
Compound	Quintile 1	Quintile 2	Quintile 3	Quintile 4	Quintile 5	***P***_trend_
Acrylamide	1 (Referent)	—^*a*^	1.02 (0.94, 1.10)	1.09 (1.02, 1.17)	1.08 (1.01, 1.16)	0.008
Acrylonitrile	1 (Referent)	1.03 (0.95, 1.12)	1.02 (0.94, 1.11)	1.05 (0.97, 1.14)	1.06 (0.97, 1.15)	0.17
Benzene	1 (Referent)	1.09 (1.00, 1.18)	1.03 (0.95, 1.12)	1.03 (0.95, 1.12)	1.06 (0.98, 1.16)	0.38
Benzidine	1 (Referent)	—^*a*^	0.98 (0.86, 1.12)	0.97 (0.91, 1.04)	1.06 (0.99, 1.14)	0.24
1,3-Butadiene	1 (Referent)	0.98 (0.91, 1.07)	1.06 (0.98, 1.15)	0.99 (0.91, 1.08)	1.02 (0.94, 1.11)	0.56
Carbon tetrachloride	1 (Referent)	0.98 (0.90, 1.07)	1.04 (0.96, 1.13)	1.03 (0.95, 1.12)	1.08 (1.00, 1.18)	0.03
Chloroprene	1 (Referent)	—^*a*^	—^*a*^	1.05 (0.96, 1.15)	1.07 (1.00, 1.14)	0.04
1,4-Dioxane	1 (Referent)	1.04 (0.96, 1.13)	1.05 (0.96, 1.14)	1.07 (0.99, 1.16)	1.02 (0.94, 1.11)	0.23
Ethyl carbamate	1 (Referent)	—^*a*^	—^*a*^	0.97 (0.90, 1.05)	1.07 (1.00, 1.14)	0.22
Ethylene dibromide	1 (Referent)	1.05 (0.97, 1.14)	1.07 (0.99, 1.16)	1.03 (0.95, 1.12)	1.01 (0.93, 1.10)	0.88
Ethylene dichloride	1 (Referent)	1.04 (0.95, 1.12)	0.94 (0.86, 1.02)	1.04 (0.96, 1.13)	1.05 (0.97, 1.14)	0.25
Ethylene oxide	1 (Referent)	0.93 (0.85, 1.00)	0.92 (0.85, 1.00)	0.97 (0.89, 1.05)	1.00 (0.92, 1.08)	0.70
Ethylidene dichloride	1 (Referent)	1.01 (0.93, 1.10)	1.09 (1.00, 1.18)	1.08 (0.99, 1.17)	1.02 (0.94, 1.11)	0.19
Hydrazine	1 (Referent)	—^*a*^	0.92 (0.86, 0.99)	0.98 (0.91, 1.06)	1.04 (0.97, 1.12)	0.36
Methylene chloride	1 (Referent)	0.97 (0.89, 1.05)	1.06 (0.98, 1.15)	1.01 (0.93, 1.10)	1.04 (0.96, 1.13)	0.21
4,4'-Methylene bis(2-chloroaniline)	1 (Referent)	—^*a*^	—^*a*^	1.02 (0.92, 1.13)	1.07 (1.01, 1.15)	0.03
Nitrobenzene	1 (Referent)	—^*a*^	—^*a*^	1.04 (0.97, 1.12)	1.03 (0.96, 1.10)	0.29
Propylene dichloride	1 (Referent)	1.00 (0.92, 1.08)	0.92 (0.85, 1.01)	1.01 (0.93, 1.09)	1.04 (0.96, 1.13)	0.20
Propylene oxide	1 (Referent)	1.05 (0.97, 1.15)	1.11 (1.02, 1.20)*	1.05 (0.97, 1.14)	1.01 (0.93, 1.10)	0.18
Styrene	1 (Referent)	1.04 (0.96, 1.13)	1.02 (0.94, 1.11)	1.05 (0.96, 1.14)	1.04 (0.96, 1.13)	0.41
2,4-Toluene diisocyanate	1 (Referent)	1.05 (0.96, 1.14)	1.04 (0.96, 1.13)	1.03 (0.95, 1.12)	1.07 (0.98, 1.16)	0.17
o-Toluidine	1 (Referent)	—^*a*^	—^*a*^	1.10 (1.01, 1.21)	1.03 (0.97, 1.10)	0.10
Vinyl chloride	1 (Referent)	1.03 (0.94, 1.12)	1.12 (1.03, 1.21)*	1.07 (0.99, 1.17)	1.06 (0.98, 1.16)	0.06
Vinylidene chloride	1 (Referent)	0.97 (0.90, 1.06)	0.98 (0.90, 1.07)	1.04 (0.96, 1.13)	1.03 (0.94, 1.11)	0.27
Summary variable	1 (Referent)	0.98 (0.90, 1.07)	0.97 (0.89, 1.05)	1.02 (0.94, 1.10)	1.05 (0.96, 1.14)	0.11

Models stratified by age and adjusted for race. Quintiles based on distribution of all study participants.

^*a*^Quintiles combines when a larger portion of the study participants had same concentration value.

*Remains statistically significant (p < 0.05) after adjustment for multiple comparisons.

Select results by tumor hormone responsiveness and for subset populations are presented in Table 3. Only those results that remained statistically significant after adjustment from multiple comparisons are presented in this table. When we restricted our analyses to breast cancer tumors that were either estrogen-receptor or progesterone-receptor positive (ER+/PR+), the overall pattern of estimated risks was generally similar to those observed for all tumor types considered in our original analysis (data not shown). For these tumor types, risks appeared to be marginally stronger than those seen for all tumor types combined (i.e., larger and statistically significant HRs) for a number of compounds including: acrylamide, benzidine, carbon tetrachloride, ethylidene dichloride, and vinyl chloride. While initial models suggested a monotonic exposure response (p-trend <0.05) for all these compounds, after adjusting for multiple comparisons, the trend remained significant only for carbon tetrachloride, for which the HR in the top quintile was 1.13 (95% CI: 1.03-1.25). In contrast, an increased breast cancer risk associated with benzene was only seen among tumors that were both estrogen-receptor negative and progesterone-receptor negative (ER-/PR-) for which a HR of 1.45 was observed for the highest quintile of benzene concentration.

**Table 3:** **HRs (95**% CIs) for breast cancer incidence by quintile of estimated exposure among subsets for select** individual mammary gland carcinogenic compounds

Compound	Subset	N cases	Quintile of concentration					
			Quintile 1	Quintile 2	Quintile 3	Quintile 4	Quintile 5	P _trend_
Acrylamide	ER+/PR+	4,352	1 (Referent)	—^*a*^	1.01 (0.92, 1.11)	1.15 (1.06, 1.24)*	1.04 (0.96, 1.12)	0.020
Benzene	ER-/PR-	704	1 (Referent)	1.24 (0.97, 1.57)	1.10 (0.86, 1.41)	1.02 (0.79, 1.31)	1.45 (1.15, 1.83)*	0.016
Benzidine	ER+/PR+	4,352	1 (Referent)	—^*a*^	0.97 (0.83, 1.13)	1.00 (0.92, 1.07)	1.12 (1.03, 1.20)*	0.022
	BMI <25	3,123	1 (Referent)	—^*a*^	0.96 (0.80, 1.16)	1.01 (0.92, 1.10)	1.19 (1.09, 1.30)*	0.001*
Carbon tetrachloride	ER+/PR+	4,352	1 (Referent)	1.03 (0.93, 1.13)	1.10 (1.00, 1.21)	1.11 (1.01, 1.22)	1.13 (1.03, 1.25)*	0.020*
	Postmenopausal	3,604	1 (Referent)	0.96 (0.86, 1.07)	1.07 (0.97, 1.19)	1.01 (0.91, 1.12)	1.13 (1.02, 1.26)*	0.004*
	HT past/never	1,052	1 (Referent)	0.96 (0.79, 1.18)	1.04 (0.85, 1.26)	1.01 (0.83, 1.23)	1.25 (1.04, 1.52)*	0.003*
	BMI <25	3,123	1 (Referent)	1.07 (0.96, 1.20)	1.11 (0.99, 1.24)	1.05 (0.94, 1.18)	1.18 (1.05, 1.32)*	0.012*
Chloroprene	BMI ≥25	2,311	1 (Referent)	—^*a*^	—^*a*^	1.04 (0.91, 1.20)	1.16 (1.05, 1.27)*	0.006
Ethyl carbamate	BMI ≥25	2,311	1 (Referent)	—^*a*^	—^*a*^	1.03 (0.93, 1.15)	1.16 (1.06, 1.28)*	0.008
Ethylidene dichloride	ER+/PR+	4,352	1 (Referent)	1.05 (0.95, 1.16)	1.20 (1.09, 1.32)*	1.15 (1.05, 1.27)*	1.05 (0.95, 1.16)	0.029
	HT past/never	1,052	1 (Referent)	1.09 (0.89, 1.34)	1.18 (0.96, 1.44)	1.35 (1.11, 1.64)*	1.28 (1.05, 1.56)	0.002*
4,4'-Methylene bis(2-chloroaniline)	BMI ≥25	2,311	1 (Referent)	—^*a*^	—^*a*^	1.13 (0.97, 1.31)	1.15 (1.05, 1.27)*	0.002*
Propylene oxide	Pre/ perimenopausal	1,548	1 (Referent)	1.19 (1.01, 1.40)	1.23 (1.05, 1.44)*	1.22 (1.04, 1.43)	1.15 (0.97, 1.35)	0.012
	BMI <25	3,123	1 (Referent)	1.09 (0.98, 1.22)	1.16 (1.04, 1.29)*	1.04 (0.93, 1.17)	1.06 (0.95, 1.19)	0.116
Vinyl chloride	ER+/PR+	4,352	1 (Referent)	1.06 (0.96, 1.17)	1.23 (1.12, 1.35)*	1.14 (1.03, 1.25)*	1.08 (0.98, 1.19)	0.021
	Postmenopausal	3,604	1 (Referent)	1.04 (0.94, 1.16)	1.14 (1.02, 1.26)*	1.08 (0.97, 1.20)	1.06 (0.95, 1.18)	0.143
	HT past/never	1,052	1 (Referent)	1.05 (0.85, 1.28)	1.21 (0.99, 1.47)	1.34 (1.10, 1.63)*	1.27 (1.04, 1.54)	0.002*
	HT current	2,204	1 (Referent)	1.11 (0.97, 1.27)	1.17 (1.03, 1.34)*	1.00 (0.87, 1.15)	1.01 (0.88, 1.16)	0.991

Models stratified by age and adjusted for race. Quintiles based on distribution of all study participants.

^*a*^Quintiles combines when a larger portion of the study participants had same concentration value.

*Remains statistically significant (p < 0.05) after adjustment for multiple comparisons.

**HRs for no other compounds remained statistically significant in any other subset analysis after adjustment for multiple comparisons.

There were a number of statistically significant results for different compounds within certain subsets of the population (Table 3). For subsets based on menopausal status, elevated risks were observed for propylene oxide among pre/perimenopausal women, and for carbon tetrachloride and vinyl chloride among postmenopausal women. For subsets based on HT use, elevated risks were seen for vinyl chloride among current HT users, and for carbon tetrachloride, ethylidene dichloride, and vinyl chloride among past/never HT users. Lastly, for subsets based on BMI, elevated risks were observed for benzidine, carbon tetrachloride, and propylene oxide among women with BMI <25, and for chloroprene, ethyl carbamate, and 4,4′-methylene bis(2-chloroaniline) among women with BMI ≥25.

For those 18 compounds with tobacco smoke as a potential source, analyses among non-smokers were generally similar to the initial analyses (data not shown). The only large change observed was among o-toluidine exposure, for which the HR in the third quintile increased to 1.17 (95% CI: 1.05-1.31) compared to the main analysis.

Among the subset of participants who were not known to have moved during the follow-up period, we found that results were generally similar to the initial analysis (data not shown). Only one of the two initially significant sets of results in the main analysis remained statistically significant among the non-mover subpopulation. For propylene oxide, the pattern of the exposure-response relation was similar, though slightly more elevated, in non-movers compared to the main analysis, and there was a statistically significant HR of 1.16 among women in the third quintile (95% CI: 1.05-1.29).

### Risk analysis based on summary MGC variable

Results using the summary MGC variable as the exposure metric displayed a positive trend with participants in the highest quintile having a HR of 1.05 for breast cancer incidence compared with those in the in the lowest quintile (Table 2). None of these results, however, were statistically significant, nor was the test for trend. Among the subsets examined and outcomes by tumor hormone responsiveness, no statistically significant results were observed (data not shown).

## Discussion

Overall, we observed little evidence of risk associated with ambient exposure to MGC HAPs. While our initial models yielded some elevated risk estimates, after adjusting for multiple comparisons and breast cancer risk factors, confidence intervals were broader, as would be expected, and tended to include one. Our subset analyses, however, suggest there may be interesting elevations in risk associated with some compounds for certain subpopulations of women and/or types of breast cancer tumors.

The subset analyses were designed to assess the degree to which risk associations might be evident for breast cancer subtypes and risk groups in which there traditionally has been evidence for differential effects for personal and lifestyle risk factors [36, 37]. It is interesting to note that some of the suggestive risk associations for MGCs appeared to be somewhat stronger (e.g., acrylamide, carbon tetrachloride, and propylene oxide) and some that seemed otherwise null appeared to be elevated (e.g., benzidine and ethylidene dichloride) in the subsets of women with the more commonly occurring hormone responsive positive tumors (ER+/PR+), and among those that might be characterized with lower levels of endogenous estrogen (e.g., leaner women, postmenopausal women, and never/past HT users). Should there be a true risk association for these chemicals associated with a hormonal pathway, it is conceivable that it could be more easily observable among the backdrop of lower endogenous hormone levels. The very striking converse association for benzene, which did not appear to be associated with breast cancer risk overall, but for which there was evidence for elevated risk, significant for trend, in the smaller subset of women with hormone responsive negative (ER-/PR-) tumors suggests the potential for a very different pathway. Although patterns of risk by subset are far from clear, and could simply be artifactually due to multiple testing, they do offer some avenues for further consideration. These results, while interesting, must be interpreted cautiously.

Despite the many comparisons in this study, there appear to be a few compounds for which the results are compelling in the context of the existing toxicological and epidemiologic literature, and considering the consistency of observed patterns in risk, as well as the likely relative contribution of ambient exposures to total exposures in human populations. These are discussed in detail below.

### Carbon tetrachloride

Our results showed that those women in the top quintile of exposure for carbon tetrachloride had the highest risk for breast cancer incidence and the exposure-response had a generally positive trend. This was consistent when examining risk among the different subsets and for all subset analyses presented in Table 3 the trend remained statistically significant after adjustment for multiple comparisons.

Although there are a number of sources, the majority of carbon tetrachloride in the environment is due to direct release to the atmosphere during production, disposal, or use of the compound [38]. It is volatile at ambient temperatures, thus most of the carbon tetrachloride in the environment exists in the air, rather than in the water or soil. Furthermore, while ubiquitous in the ambient air, concentrations are somewhat higher in urban areas and near industrial sources. The general population is most likely to be exposed to carbon tetrachloride primarily via ambient air, and may also be exposed through drinking water, though at lower levels [38]. Given these sources of exposure, the NATA ambient concentrations estimates used in the models may be a reasonable proxy of inhalation exposure among study participants.

While carbon tetrachloride has been associated with mammary gland tumors in laboratory animals, few studies exist examining this association in humans. A previous case control study that relied upon qualitative measures of exposure found an association between breast cancer mortality and level of carbon tetrachloride exposure [23]. The highest risk observed was an odds ratio (OR) of 1.32 (95% CI: 1.1-1.6) among black women exposed to the third level of exposure (scale was zero to four exposure levels), compared to those in the lowest exposure category. Among white women in the study, those in the fourth level of exposure had an OR of 1.21 (95% CI: 1.1-1.3). A study of aircraft maintenance workers found that women exposed to carbon tetrachloride had an elevated rate ratio of 1.3 for breast cancer mortality, compared to women with no exposure [39]. However, there were only 18 cases and the 95% CI included the null. An ecological study by Coyle et al. found no difference in median average annual age-adjusted breast cancer rates between counties with and without reported releases of carbon tetrachloride [19]. Overall and including this study, the results are suggestive of carbon tetrachloride being associated with increased risk of breast cancer and warrant further investigation.

### Ethylidene dichloride and vinyl chloride

The exposure-response patterns for ethylidene dichloride and vinyl chloride were similar for initial and subset models, and exposure concentrations for these two chemicals were highly correlated among our study participants (Pearson correlation coefficient: 0.99). In the initial and some of the subset models, the exposure-response was an inverse-U shape, with peak risk associated with one or both middle quintiles of estimated exposure. Only for those in the past/never HT use subset was the risk consistently high for all three top quintiles of exposure, although peak risk was observed for the fourth quintile of exposure, rather than the fifth quintile. This non-monotonic risk relationship may be due to a number of reasons. First, this could be due to exposure misclassification. If there were truly a monotonic exposure-response relationship, and those who were truly in the highest exposure level were misclassified as being in the middle exposure levels, it would appear that those in the middle exposure levels had the greatest risk of breast cancer risk. Alternatively, there may be a biological explanation for this pattern. Several mechanisms have been proposed for non-monotonic dose–response curves, including cytotoxicity (cell death at high doses), receptor selectivity (differences in receptor affinity at low vs. high doses), receptor down-regulation and desensitization, and receptor competition [40, 41]. Toxicological studies of these specific compounds and the appropriate receptors at relatively low exposure levels such as those estimated in the current study would be needed in order to determine the likely underlying mechanism.

The concordance of results and high correlation between these two chemicals is not unexpected given that ethylidene dichloride is an intermediate in the production of vinyl chloride, which, in the U.S., is almost exclusively produced to make polyvinyl chloride (PVC). Anthropogenic sources are responsible for all of the ethylidene dichloride and vinyl chloride found in the environment, most of which is released from industrial processes almost entirely to the atmosphere [42, 43]. Both compounds volatilize into the air; therefore the most likely route of exposure is via inhalation. Exposure to these chemicals for the general population is primarily through inhalation of contaminated air, especially near emission source areas. Other potential routes of exposure are ingestion of contaminated drinking water and use of consumer products that contain these chemicals [42, 43].

There are no published epidemiologic studies specifically examining exposure to either ethylidene dichloride or vinyl chloride and risk of breast cancer incidence or mortality. There have been, however, two reports on workers in PVC manufacturing. A cross-sectional mortality study among employees of 17 PVC fabricators found a statistically significantly elevated risk of breast cancer mortality among white female workers (proportionate mortality ratio: 1.37) [44], consistent with an earlier report from the same cohort [45], though there were only 44 breast cancer deaths in this population. Further, a recent review of the literature by the Institute of Medicine stated that there is considerable animal evidence indicating the biologic plausibility of the potential for induction of breast cancer from vinyl chloride [46]. Our results in the context of this very limited human health evidence are provocative and suggest the need for future research to elucidate the relationship between exposure to ethylidene dichloride and/or vinyl chloride and risk of breast cancer. Our results further suggest such research should consider these exposures in the context of hormonal exposures and tumor hormone responsiveness.

### Benzene

Our results suggest that exposure to benzene may only increase breast cancer risk for ER-/PR- breast tumors. Benzene is widely used and ranks among the top 20 for production volume for chemicals produced in the United States. Industrial processes are the main sources of benzene in the environment, and ambient air concentrations can also be elevated by emissions from burning coal and oil, motor vehicle exhaust, and benzene waste and gasoline storage operations [47]. Because benzene partitions mainly into air, inhalation is the dominant pathway of human exposure accounting for >99% of the total daily intake of benzene. The general population is exposed primarily via tobacco smoke and by inhaling contaminated air. Among non-smokers the major sources of exposure are traffic-related air pollution and gasoline [47]. There are only two other studies that have examined female breast cancer risk and at minimum semi-quantitative measures of benzene exposure. A study of benzene exposure and breast cancer mortality and incidence among female shoe factory workers in Italy found elevated, but non-significant, risk associations [48]. The standardized mortality ratio was most elevated for women with >40 ppm-years of cumulative exposure with ≥30 years of latency, at 166.0 (95% CI: 62.3-442.2) based on four deaths. The standardized incidence ratio (SIR) was most elevated for women with >40 ppm-years of cumulative exposure with <30 years of latency, at 211.9 (95% CI: 29.9-1504) based on only one case. SIRs, however, were also elevated among women with ≤40 ppm-years of cumulative exposure with <30 years of latency (135.8 (95% CI: 70.7-261.1), based on nine cases), and among women with >40 ppm-years of cumulative exposure with ≥30 years of latency (122.3 (42.4-245.0), based on 6 cases). A case–control study conducted by Petralia and colleagues [22] using job histories and a job-exposure matrix based on occupation and industry codes found a significant overall increased risk of premenopausal breast cancer associated with ever being occupationally-exposed to benzene (OR: 1.70; 95% CI: 1.17-2.92). When separated into ER+ and ER- tumor types, the OR for ever occupationally-exposed to benzene increased to 2.20 for ER- breast cancer (n cases = 12), although this result was no longer statistically significant (95% CI: 0.087-5.53). Given the consistency of these findings with our own, and the widespread human exposures to benzene, further research is warranted. Our results further suggest that such evaluations should be conducted in study populations with sufficient numbers of breast cancer cases to enable a targeted evaluation of risk for ER-/PR- breast cancer.

### Limitations and strengths

In our study we examined risk from modeled ambient concentrations for a large number of MGC compounds. We conducted analyses based upon individual compounds, but we also attempted to account for exposure to multiple pollutants by using a summary MGC variable. The results showed that the overall summary variable was not statistically significantly associated with increased risk. There may be a number of reasons for this. First, including compounds with no risk association in the summary variable may dilute the effect of those compounds with an association. Second, if total concentration is important, scaling each compound to a standard distribution (mean set to zero and standard deviation to one) before summing across compounds would dilute the summary effect. Finally, the individual compound models may have been more likely to detect signals in exposure data than the summary variable models because the latter summed across multiple distributions of compounds, producing an exposure with a lower signal-to-noise ratio. While we have examined only one type of summary measure, other summary measures using different summation methods remain worth exploring.

There were some limitations in this study. Perhaps foremost is the potential for exposure misclassification. Residential addresses at the baseline survey (1995–96) were linked with census tract level estimates of annual average ambient concentrations for the year 2002. We assumed that the relative concentrations were representative of the entire follow-up period. Our selection of the 2002 concentration estimates were based on a previous study examining agreement over time between NATA estimates and monitored data in California [34]. Although there may be concentration changes over time, we felt the 2002 data offered the best option available as it was mid-way through the follow-up period. To assess the impact of exposure misclassification due to exposure estimates being based solely on baseline address, we analyzed data based on a subset of participants who had no record of moving during the follow-up period and found that results were similar to those from the main analysis. There were about 10% more movers among the non-cases compared with the cases, suggesting the potential for differential exposure misclassification. However, because the overall pattern for HR exposure-response remained generally similar to that seen in our full study population, we do not consider exposure misclassification to be a substantial source of bias in our study.

Second, our analyses are predicated on the assumption that these modeled ambient concentrations can serve as reasonable proxies for inhalational exposure to these compounds, which may not be the case. The estimated concentrations provided by the EPA are based on complex modeling of emissions data, subject to the constraints of the data and the assumptions underlying the predictive models. The EPA provides an overall confidence in exposure assessment rating for each compound whose concentration they estimated [49]. Of the 24 compounds examined here, four were rated “high”, nine as “medium”, and 11 were considered to have “low” confidence. Of the compounds for which we found statistically significant results, only benzene and propylene oxide were rated as high confidence, those with low confidence included acrylamide, chloroprene, and 4,4′-methylene bis(2-chloroaniline), and the remainder were of medium confidence. Moreover, these modeled annual ambient concentrations are applied to the entire census tract, with no account for intra-census tract concentration. If ambient concentrations within a census tract are highly variable, these census-tract wide averages would lead to exposure misclassification, albeit nondifferential.

Third, we could not consider indoor inhalation exposures or ambient exposures outside of the census tract of baseline residence. While the majority of the chemicals examined do not have major indoor sources, the few exceptions include benzene, 2,4-toluene diisocyanate, propylene oxide, and styrene which are likely indoor air contaminants for the general public primarily through vehicle exhaust from an attached garage or through consumer products [50–53]. If for these chemicals, indoor exposure comprises a large portion of total inhalational exposure, then the exposure estimates used in this study would underestimate the true total exposure. Likewise, some of these compounds, such as benzene and ethylene oxide [47, 54], are also found in cigarette smoke and although we did control for smoking status in the fully-adjusted models, tobacco exposures could easily overwhelm the more modest levels in ambient air. It is notable, however, that the effects we observed for benzene were stronger when we confined the analyses to never smoking non-movers.

Lastly, this study examined the risk associated only with inhalational exposure; other routes of exposure may be as, or more, important for certain chemicals. Ingestion from dietary sources is thought to be the primary or one of the major routes of exposure among the general population for acrylamide, ethyl carbamate, o-toluidine, and vinylidene chloride [55–58]. Ingestion of contaminated drinking water is thought to be a likely route of exposure for ethylene dibromide, 1,4-dioxane, nitrobenzene, and vinylidene chloride [58–61]. For these compounds, exposure via inhalation may be low for some individuals compared to these other routes of exposure.

Despite these limitations, this study offers a number of advances over previous research on air pollution and breast cancer risk. First, these analyses use data from a large prospective cohort of women with the opportunity to account for covariate information on individual breast cancer risk factors. While the individual SES of study participants was not assessed, it is likely to be fairly homogeneous within this study population by virtue of the CTS being an occupational cohort of professional women, all of whom have at least a 4-year college degree. Furthermore, the incorporation of adjustment for a summary measure of neighborhood SES further reduces the likelihood that our results are merely a reflection of residual confounding due to SES, although we cannot fully dismiss that possibility. Additionally, because of the longitudinal nature of this study misclassification of menopausal status is a concern for women who reported being pre-/peri-menopausal at baseline (43%). This misclassification would have affected primarily the stratified analyses among pre-/peri-menopausal women, pooling together women who were still pre-/peri-menopausal with those who had become menopausal. If pre-/peri-menopausal women where truly more susceptible to the effects of these exposure than menopausal women, results would have been biased towards null due to this misclassification. Previous studies have relied on cancer registry data [19] or on occupational cohorts that contain little or no information on personal-level risk factors. Second, we used a more granular level of quantitative exposure estimates to these hazardous air pollutants. The only other study with quantitative exposure estimates, was an ecologic study based on reported emission releases for six chemicals and six metals obtained from the EPA Toxic Release Inventory [19]. But the county-level scale of that study and crude dichotomy of presence or absence of reported releases reduced specific interpretability. Most other studies on this topic have used data from limited air monitoring stations or a more qualitative exposure assessment such as exposed/not exposed, low/high exposure potential, or job category [20–22, 25, 26].

## Conclusions

Using pre-existing EPA data on annual average ambient concentrations of MGC hazardous air pollutants at the census tract level, we found statistically significant associations between increased risk of breast cancer and residence in areas with high estimated ambient concentrations of propylene oxide and vinyl chloride. Suggestive evidence of an association with breast cancer incidence was also seen in certain subpopulations for several MGCs, most notably for carbon tetrachloride, ethylidene dichloride, and vinyl chloride. Stratified by tumor hormone responsiveness, ER+/PR+ tumors were associated with estimated exposure to acrylamide, benzidine, carbon tetrachloride, ethylidene dichloride, and vinyl chloride, while ER-/PR- tumors were associated with estimated benzene exposure. Additionally, we found that using a summary variable for all 24 MGCs of interest was not fruitful and did not offer any advantage over the individual compound models. This is the first study to quantitatively examine the relationship between ambient residential exposures to select hazardous air pollutants and risk of breast cancer incidence among women using individual-level data. Risk relationships for exposures to these MGCs should be further examined.

## Consent

Participants of this study are all members of the CTS cohort who provided written informed consent at the time they joined the cohort. This consent included consent to be contacted in the future for follow-up data collection, consent to allow their data to be linked to the California Cancer Registry and consent for their data to be used for research purposes.

## Electronic supplementary material

Additional file 1: Table S1: Select information for 24 Mammary Gland Carcinogens. (PDF 21 KB)

## Abbreviations

95% CI: 95% Confidence intervals
ASPEN: Assessment System for Population Exposure Nationwide
BMI: Body mass index
CCR: California Cancer Registry
CTS: California Teacher Study
EPA: United States Environmental Protection Agency
HAPs: Hazardous Air Pollutants
HEM: Human Exposure Model
HR: Hazard rate ratio
HT: Hormone therapy
MGCs: Mammary gland carcinogens
NATA: National-Scale Air Toxics Assessment
OR: Odds ratio
PVC: Polyvinyl chloride
SES: Socioeconomic status
SIR: Standardized incidence ratio.
