# Supplementary material for: Hazardous air pollutants and breast cancer risk in California teachers: a cohort study
Source: Environ Health. 2015 Jan 30;14:14. doi: 10.1186/1476-069X-14-14 (PMC4417287; doi:10.1186/1476-069X-14-14)
Supplement: Supplementary file 1 — Additional file 1: Table S1: Select information for 24 Mammary Gland Carcinogens. (PDF 21 KB) [file 12940_2014_848_MOESM1_ESM.pdf]

Supplementary Table S1: Select information for 24 Mammary Gland Carcinogens examined in the analysis.

Compound	*Probable Routes of Human Non-Occupational Exposure* [a]	*Predominant Source Type for NATA-modeled Exposure among Our Study Population* [b]	*IARC Classification* [c]	*Overall Confidence in Exposure Assessment* [d]
Acrylamide	-Ingestion of certain foods that have been cooked and processed at high temperature<br>-Tobacco smoke<br>-Ingestion of contaminated drinking water<br>-Dermal contact with polyacrylamide products	Major	2A	Lower
Acrylonitrile	-Inhalation of ambient air, particularly near industrial plant manufacturing/using acrylonitrile<br>-Dermal contact with products containing acrylonitrile<br>-Tobacco smoke	Background	2B	Higher
Benzene	-Inhalation of ambient air, particularly in areas with heavy vehicular traffic, gasoline stations, and industrial plants manufacturing/using benzene<br>-Ingestion of food and drinking water<br>-Tobacco smoke<br>-Dermal contact with consumer products containing benzene<br>-Use and storage of gasoline and gas-powered tools	On-road	1	Higher
Benzidine	-General population not thought to be exposed; benzidine may only be produced for captive use in the United States.<br>-Tobacco smoke	Background	1	Lower
1,3-Butadiene	-Inhalation of ambient air, particularly in areas with heavy vehicular traffic and manufacturing and processing facilities<br>-Ingestion of drinking water<br>-Dermal contact directly with 1,3-butadiene or products containing this compound<br>-Tobacco smoke	On-road	1	Lower
Carbon tetrachloride	-Inhalation of ambient air<br>-Indoor air, particularly from building materials or products used in home<br>-Ingestion of contaminated foods and drinking water<br>-Dermal contact with carbon tetrachloride and products containing this compound<br>-Tobacco use	Background	2B	Medium
Chloroprene	-Inhalation of ambient air	Major	2B	Lower
1,4-Dioxane	-Inhalation of ambient air<br>-Ingestion of drinking water<br>-Dermal contact with contaminated consumer products	Major	2B	Medium
Ethyl carbamate	-Ingestion of fermented foods and alcoholic beverages<br>-Ingestion of drinking water<br>-Tobacco smoke	Major	2A	Medium
Ethylene dibromide	-Inhalation of ambient air<br>-Ingestion of drinking water<br>-Tobacco smoke	Background	2A	Medium
Ethylene dichloride	-Inhalation of ambient air<br>-Ingestion of drinking water<br>-Dermal contact with ethylene dichloride or consumer products containing this compound<br>-Tobacco smoke	Background	2B	Medium
Ethylene oxide	-Inhalation of ambient air, particularly near areas of heavy vehicular traffic and industrial facilities<br>-Tobacco smoke	Background	1	Higher
Ethylidene dichloride	-Inhalation of ambient air, particularly near source areas<br>-Ingestion of contaminated drinking water<br>-Use of consumer products that contain ethylidene dichloride	Major	3	Medium

Supplementary Table S1: Select information for 24 Mammary Gland Carcinogens examined in the analysis.

Compound	*Probable Routes of Human Non-Occupational Exposure* [a]	*Predominant Source Type for NATA-modeled Exposure among Our Study Population* [b]	*IARC Classification* [c]	*Overall Confidence in Exposure Assessment* [d]
Hydrazine	-Tobacco smoke<br>-Ingestion of food<br>-Dermal contact with vapors and other products containing hydrazine	Major	2B	Lower
Methylene chloride	-Inhalation of ambient air<br>-Indoor air, particularly from consumer products<br>-Ingestion of food and drinking water<br>-Dermal contact with consumer products containing methylene chloride<br>-Tobacco smoke	Area	2B	Higher
4,4'-Methylene bis(2-chloroaniline)	-Inhalation of ambient air, particularly near industrial plant manufacturing/using 4,4'-Methylene bis(2-chloroaniline)<br>-Dermal contact with compound in vicinity of production and manufacturing facilities	Major	1	Lower
Nitrobenzene	-Inhalation of ambient air<br>-Ingestion of drinking water<br>-Dermal contact<br>-Tobacco smoke	Major	2B	Lower
Propylene dichloride	-Inhalation of ambient air<br>-Ingestion of drinking water<br>-Dermal contact with this propylene dichloride and other products containing this compound<br>-Tobacco smoke	Background	1	Lower
Propylene oxide	-Inhalation of ambient air<br>-Ingestion of contaminated food<br>-Use of consumer products<br>-Tobacco smoke	Major	2B	Higher
Styrene	-Indoor air, particularly from building materials and consumer products<br>-Ingestion of foods packaged in polystyrene<br>-Ingestion of contaminated drinking water<br>-Inhalation of ambient air, particularly near industrial sources, heavy vehicular traffic, and incineration emissions<br>-Tobacco smoke<br>-Use of consumer products	Major	2B	Lower
2,4-Toluene diisocyanate	-Use of consumer products containing this compound<br>-Inhalation of contaminated ambient air	Major	2B	Lower
o-Toluidine	-Ingestion of food<br>-Tobacco smoke<br>-Dermal contact with o-toluidine or other products containing this compound	Major	1	Lower
Vinyl chloride	-Inhalation of ambient air, particularly near industrial sources<br>-Ingestion of food and drinking water<br>-Tobacco smoke	Major	1	Medium
Vinylidene chloride	-Inhalation of ambient air, particularly near industrial sources<br>-Ingestion of food and drinking water<br>-Dermal contact with vinylidene chloride or other products containing this compound	Major	3	Medium

[a]Abstracted from the *Hazardous Substances Database* (http://toxnet.nlm.nih.gov/newtoxnet/hsdb.htm) and the US EPA Air Toxics Web Site *Health Effects Notebook for Hazardous Air Pollutants*

[b]See NATA glossary of terms (http://www.epa.gov/ttn/atw/nata2002/gloss.html)

[c]Obtained from Agents Classified by the *IARC Monographs* , Volumes 1-11 (http://monographs.iarc.fr/ENG/Classification/ClassificationsGroupOrder.pdf)

[d]Provided by US EPA as part of NATA documentation (http://www.epa.gov/ttn/atw/nata2002/02pdfs/2002polls.pdf)
